# Modelling Like an Experimentalist

**DOI:** 10.1111/ele.70251

**Published:** 2025-11-05

**Authors:** John M. Drake

**Affiliations:** ^1^ Odum School of Ecology University of Georgia Athens Georgia USA; ^2^ Center for the Ecology of Infectious Diseases University of Georgia Athens Georgia USA

**Keywords:** experimental design, modelling, pedagogy

## Abstract

Modelling has become a routine part of ecological and evolutionary research, yet its practice often lacks a clear conceptual framework. I propose that modelling can be fruitfully understood as experimentation. Like empirical studies, modelling projects involve treatments, levels and responses: parameter regimes or data manipulations serve as treatments, replicated runs yield summaries and comparisons across conditions reveal main effects and interactions. This framing sharpens design, reduces mission creep and clarifies communication. I outline common layers of abstraction, highlight good modelling habits and argue that thinking like an experimentalist fosters rigour, reliability and credibility without constraining creativity.

## Graphical abstract

Dahlin et al. (2024) apply experimental thinking to a model of mosquito‐borne disease transmissions.

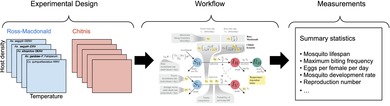

When I was in graduate school in the late 1990s and early 2000s, modellers were a specialised species occupying a relatively narrow niche. Modelling demanded mathematics more advanced than most ecologists encountered in their training and typically required writing custom computer code—whether for numerical analysis of dynamical systems, agent‐based simulations or bespoke statistical routines. Statistical analysis, by contrast, was dominated by packaged procedures, most often SAS ‘Procs’, a menu‐driven framework in which the researcher specified a model and the software handled the heavy lifting. Modelling, in other words, was for the few.

A quarter century later, the situation looks very different. Sophisticated pipelines for analysing massive datasets—whether remotely sensed environmental variables, high‐throughput genomic sequences or long‐term sensor streams—are now part of the working toolkit for many researchers in ecology and evolutionary biology. Tools for numerical analysis are now widely available and well supported. Programming literacy is viewed as vital and growing in significance (Farrell and Carey 2018). I suspect that today it would be hard to find an ecologist or evolutionary biologist whose research does not involve coding in some form.

Survey evidence underscores this transformation. A 2013 study reported that in mainstream ecological journals the majority of papers already employed statistical and computational techniques well beyond ANOVA and simple regression (Barraquand et al. 2014). In their survey of July 2012 issues, 75% of articles in *Ecology*, 95% in the *Journal of Animal Ecology* and 70% in *Oikos* used advanced quantitative methods such as generalised or mixed regression models, maximum likelihood, Bayesian inference with MCMC, graph‐theoretic algorithms or stochastic movement models. Nearly all respondents to their survey (96%) reported using mathematics for statistics, and substantial fractions also used mathematics for theoretical modelling (39%) or decision‐making (24%). Clearly, the practice of modelling has become mainstream. We should no longer talk about *modellers* (an identity) but *modelling* (a method for research).

Yet for all this growth, something is missing. Modelling has spread in practice faster than it has matured in concept. We have the tools to build models, but not always a clear way to think about them as a scientific process. Too often workflows are assembled ad hoc, projects drift beyond their initial aims and presentations of results in papers, talks or proposals become tangled. What is needed is a framework that helps us plan more deliberately, avoid mission creep and communicate more clearly. Fortunately, such a framework already exists in science, and it is one that every biologist knows instinctively: experimentation.

Modelling already operates under a logic of experimentation, whether one acknowledges it or not. Parameters are varied, structures are altered, data are included or omitted, instances are replicated and outputs are compared. These are the practices of bench or field scientists who define treatments, impose controls and measure responses. The difference is only in medium: modelling experiments unfold in silico rather than in glassware or field plots. From this simple observation comes a useful reframing: modelling projects can be designed and communicated as experiments, with explicit treatments, levels and responses. Such framing sharpens design, improves interpretation, clarifies communication and reminds us that simulations are organised inquiries.

## 1 Concept 1: Two Kinds of Modelling Have Parallel Workflows

The structure of the survey by Barraquand and colleagues usefully distinguishes between two main uses of mathematics in ecology: statistical analysis and theoretical modelling. It would seem that ecologists and evolutionary biologists have two basic computational workflows: (1) The *analytical workflow* begins with data, proceeds through code and ends with a particular conclusion about the populations from which the data are drawn; (2) The *theoretical workflow* begins with ideas, proceeds through code and ends with a general conclusion about the concepts embodied in, or entailed by, those ideas.

Seen through the lens of experimentation, both workflows can be cast in the same frame. In the analytical workflow, the ‘treatments’ are data ablations (systematic omissions of parts of the dataset to test dependence on portions of the data), alternative pipelines or robustness checks, and the ‘responses’ are the fitted parameters, predictions or effect sizes. In the theoretical workflow, the ‘treatments’ are parameter regimes, simplifying assumptions or alternative model structures, and the ‘responses’ are the resulting system behaviours. In both cases, the gain is clarity: treatments are defined, responses are measured and comparisons can be drawn. And because the logic of experimental design applies equally well to both workflows, the same principles for structuring, interpreting and communicating results can guide modelling whether it is abstract or data‐driven.

What we learn when viewing workflows this way is that modelling decisions are not just background details but the experimental design itself. Special cases become treatments, variants define levels and the responses are whatever summaries or estimates the study is built to produce. Once framed this way, modelling projects are easier to plan, less prone to mission creep and clearer to present in grants, talks or manuscripts. Just as in empirical experiments, one might even examine interactions between treatments—for example, whether the effect of a contact network structure depends on the choice of transmission kernel, or whether the influence of a prior depends on the way missing data are handled.

This framing is not entirely without precedent. Similarities between experiments and simulation studies have been discussed before (Peck 2004; Railsback and Grimm 2011; Lorscheid et al. 2012), particularly in the challenging context of how to document simulation work (Grimm et al. 2006). But the broader implication, that the analogy reveals something about the logic of theory in science itself, has rarely been articulated. As an example, Dahlin et al. (2024) were interested in the theoretical issue of how vertebrate host abundance and defensive measures affect temperature dependence of mosquito‐borne parasite transmission. Their design involved four treatments: (1) *defensive measures*, with two levels (the classical Ross–Macdonald model without defence vs. the Chitnis model with defence); (2) *temperature*, varied continuously from 10°C to 40°C; (3) *host density*, varied continuously from 15 to 40 individuals per hectare; and (4) *mosquito–pathogen system*, with five levels (*Aedes aegypti*–DENV, *Aedes aegypti*–ZIKV, *Aedes albopictus*–DENV, *Anopheles gambiae*–*Plasmodium falciparum* and *Culex quinquefasciatus*–WNV). The two gradients (temperature and host density) represent treatments with an effectively infinite number of levels, something impossible in nature but easily accomplished in silico. The result is a 2 × ∞ × ∞ × 5 factorial design, high‐dimensional but entirely tractable.

**FIGURE 1:**
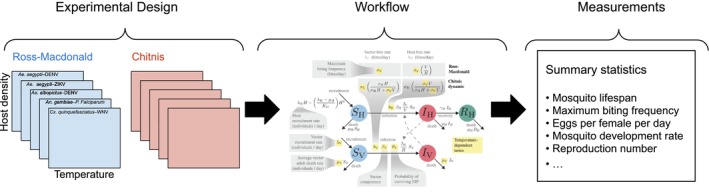
Dahlin et al. (2024) apply experimental thinking to a model of mosquito‐borne disease transmission. *Left*: Input variables representing treatments, including defensive measures (two levels), temperature (gradient), host density (gradient) and mosquito–pathogen systems (five levels). *Centre*: Schematic representation of the dynamical system model linking inputs to outcomes. *Right*: Model outputs serving as responses, including life‐history traits (e.g., development rate, survival), transmission traits (e.g., biting frequency, vector competence) and epidemiological metrics (e.g., basic reproduction number, thermal optimum). Together, these elements form a factorial design analogous to an empirical experiment.

The primary outputs measured by Dahlin et al. (2024) included adult mosquito lifespan, maximum biting frequency, eggs per female per day, mosquito development rate, the probability of immature mosquito survival, the probability of surviving the extrinsic incubation period, vector competence, basic reproduction number, thermal optimum for transmission and range in parasite thermal tolerance. By propagating variation in each input dimension through their models, Dahlin et al. (2024) generated a rich set of outputs. This is precisely what it means to treat modelling as an experiment: explicit treatments, structured levels and measured responses, all organised to address a clear theoretical question (conceptually represented in Figure 1).

Naturally, translating such a vast input space into a reader‐friendly view of the output space is no small task. Dahlin et al. (2024) manage it with multi‐panel heatmaps, a visualisation almost dictated by the factorial geometry of their design.

The same logic applies to statistical workflows. Here, the treatments might be data ablations as well as tuning parameters (such as bandwidths in smoothing), robustness checks (e.g., dropping outliers) or alternative model specifications. Each of these choices defines a treatment, with the fitted coefficients, predictive accuracy or other diagnostics serving as the responses.

## 2 Concept 2: From Outputs to Responses—Building the Layers

Just as experiments generate data at multiple levels, modelling produces outputs that can be organised in layers:

1. *Instances* (*run level*). Raw trajectories or single solutions—the analogue of individual measurements (albeit often vector‐valued or matrix‐valued rather than simple scalars).
2. *Within‐condition summaries* (*treatment level*). For each treatment/level (i.e., a fixed parameter set or model specification), instances are converted into summary metrics (e.g., equilibrium density, oscillation amplitude/period, time to extinction, fixation probability) and, when stochastic, summarised across replicates (means, variances, quantiles).
3. *Among‐condition comparisons* (*design level*). Here, we examine how treatment‐level summaries vary across levels—including main effects and interactions—via contrasts, response surfaces, heatmaps or variance decompositions.

In population biology, we typically use all three layers for stochastic models (instances → within‐condition summaries → among‐condition comparisons). In deterministic models, the run and treatment‐level summaries coincide, so practice usually collapses to two layers: direct summaries of trajectories and comparisons across treatments. Classic examples include asking whether predator–prey stability depends on the functional response (main effect) and whether that dependence changes with resource productivity (interaction) (Rosenzweig 1971); how allele fixation probability varies with selection strength and population size, and whether their joint effect is non‐additive (Kimura 1962); or whether extinction risk reflects both demographic and environmental variance and the interaction between them (Lande 1993).

This layering makes clear that model outputs are not conclusions in themselves, any more than a single plant height or assay reading is a conclusion in an experiment. They are raw material, inputs to an organised chain of abstraction. In purely theoretical work, statistical significance is beside the point; what matters are effect sizes, uncertainty from stochastic replication (Monte Carlo error) and sensitivity to assumptions. When the workflow involves data manipulations such as ablations, alternative pipelines or robustness checks, comparative statistics are appropriate at the among‐condition layer to test whether conclusions hold across these variations.

## 3 Good Habits of the Experimental Modeller

Good modelling practice begins with a well‐defined question and the selection of methods appropriate to its resolution. While recent advances in machine learning and artificial intelligence have expanded the methodological frontier, not every question benefits from being forced to fit the newest algorithm. Viewing modelling as a form of experimentation also highlights a set of good habits. These practices contribute to *rigour*, by ensuring the analysis is comprehensive rather than selective; to *reliability*, by making it easier to detect mistakes or fragile assumptions; and to *credibility*, by allowing readers to see that the work has been carried out thoroughly and with due care.

### 3.1 Validate With Simulated Truth

A first habit is to test whether an analytical pipeline can recover known conditions. This is the modelling equivalent of calibrating instruments or running a standard assay before beginning a set of experiments. By simulating data under a model where the ‘truth’ is known, and then applying the intended analysis, one can verify that the procedure produces unbiased or consistent results. Just as an experimentalist would be cautious about drawing inferences from an uncalibrated sensor, the modeller should hesitate to trust results from an unvalidated pipeline.

### 3.2 Sensitivity Analysis

Experimentalists often vary treatments systematically to test the robustness of results; modellers can do the same, not only with parameters but also with the modelling decisions themselves. Choices about initial conditions, spatial or temporal resolution, or how data are filtered can be treated as experimental factors. Sensitivity analysis exposes whether the conclusions depend narrowly on assumptions or remain stable across a reasonable range and helps to make both the choices that inevitably are made and their consequences explicit. In this sense, it plays the role of a ‘robustness check’ in empirical work, akin to testing whether results hold across different batches of reagents or different sites in a field study.

### 3.3 Perturbation Techniques

Finally, modellers can deliberately break or shuffle connections in their models or data pipelines to reveal artefacts (Grimm and Berger 2016). This practice has no perfect experimental analogue, but the spirit is close to the negative control: an intentional disruption meant to show what happens in the absence of a true signal. Examples include permuting covariates, introducing dummy variables with no intended effect, randomising network connections or shuffling trait values across taxa to test whether observed patterns still emerge. If they do, the apparent effect is likely an artefact of the pipeline rather than a property of the system.

In conclusion, thinking about modelling as experimentation does not cage creativity; it structures it, giving us better tools to ask sharper questions, present clearer answers and build cumulative science. It sharpens practice: in theoretical work by imposing discipline and preventing drift into ad hoc exploration; in analytical work by clarifying how results depend on modelling choices. And across both, it provides a shared language with empiricists. If you cannot write down your treatments and responses, you are not modelling—you are doodling.

## Author Contributions

J.D. developed the ideas reported here and wrote the manuscript.

## Data Availability

The author has nothing to report.
